# Laboratory Evolution Experiments Help Identify a Predominant Region of Constitutive Stable DNA Replication Initiation

**DOI:** 10.1128/mSphere.00939-19

**Published:** 2020-02-26

**Authors:** Reshma T. Veetil, Nitish Malhotra, Akshara Dubey, Aswin Sai Narain Seshasayee

**Affiliations:** a National Centre for Biological Sciences, Tata Institute of Fundamental Research, Gandhi Krishi Vigyan Kendra, Bengaluru, Karnataka, India; b School of Life Science, The University of Trans-Disciplinary Health Sciences & Technology (TDU), Bengaluru, Karnataka, India; University of Wyoming

**Keywords:** DNA replication, DnaA, R-loops, constitutive stable DNA replication, evolution, gene expression, head-on collision, replication-transcription conflicts

## Abstract

The bacterium Escherichia coli can initiate replication in the absence of the replication initiator protein DnaA and/or the canonical origin of replication *oriC* in a *ΔrnhA* background. This phenomenon, which can be primed by R-loops, is called constitutive stable DNA replication (cSDR). Whether DNA replication during cSDR initiates in a stochastic manner through the length of the chromosome or at specific sites and how E. coli can find adaptations to loss of fitness caused by cSDR remain inadequately answered. We use laboratory evolution experiments of *ΔrnhA-ΔdnaA* strains followed by deep sequencing to show that DNA replication preferentially initiates within a broad region located ∼0.4 to 0.7 Mb clockwise of *oriC.* This region includes many bisulfite-sensitive sites, which have been previously defined as R-loop-forming regions, and includes a site containing sequence motifs that favor R-loop formation. Initiation from this region would result in head-on replication-transcription conflicts at rRNA loci. Inversions of these rRNA loci, which can partly resolve these conflicts, help the bacterium suppress the fitness defects of cSDR. These inversions partially restore the gene expression changes brought about by cSDR. The inversion, however, increases the possibility of conflicts at essential mRNA genes, which would utilize only a minuscule fraction of RNA polymerase molecules, most of which transcribe rRNA genes. Whether subsequent adaptive strategies would attempt to resolve these conflicts remains an open question.

**IMPORTANCE** The bacterium E. coli can replicate its DNA even in the absence of the molecules that are required for canonical replication initiation. This often requires the formation of RNA-DNA hybrid structures and is referred to as constitutive stable DNA replication (cSDR). Where on the chromosome does cSDR initiate? We answer this question using laboratory evolution experiments and genomics and show that selection favors cSDR initiation predominantly at a region ∼0.6 Mb clockwise of *oriC.* Initiation from this site will result in more head-on collisions of DNA polymerase with RNA polymerase operating on rRNA loci. The bacterium adapts to this problem by inverting a region of the genome including several rRNA loci such that head-on collisions between the two polymerases are minimized. Understanding such evolutionary strategies in the context of cSDR can provide insights into the potential causes of resistance to antibiotics that target initiation of DNA replication.

## Abstract (precis)

The bacterium E. coli can replicate its DNA even in the absence of the molecules that are required for canonical replication initiation. This often requires the formation of RNA-DNA hybrid structures and is referred to as constitutive stable DNA replication (cSDR). Where on the chromosome does cSDR initiate? We answer this question using laboratory evolution experiments and genomics and show that selection favors cSDR initiation predominantly at a region ∼0.6 Mb clockwise of *oriC.* Initiation from this site will result in more head-on collisions of DNA polymerase with RNA polymerase operating on rRNA loci. The bacterium adapts to this problem by inverting a region of the genome including several rRNA loci such that head-on collisions between the two polymerases are minimized. Understanding such evolutionary strategies in the context of cSDR can provide insights into the potential causes of resistance to antibiotics that target initiation of DNA replication.

## INTRODUCTION

Canonical chromosome replication in the bacterium Escherichia coli is initiated by the specific recognition of repetitive short sequence motifs within the origin of replication *oriC* by the protein DnaA. This is followed by DNA unwinding and the synthesis of an RNA primer that can then be extended by the replicative DNA polymerase III (1). Replication proceeds bidirectionally outward of *oriC* before terminating at a locus positioned diametrically opposite *oriC* on the circular chromosome (2).

Bidirectional replication from a single *oriC* might have been the selective force behind the evolution of several organizational features of the genomes of bacteria, especially of those capable of rapid growth. These features include the encoding of highly expressed essential genes close to *oriC* to take advantage of the higher copy number of these loci while replication is in progress and on the leading strand of replication to minimize the detrimental effects of head-on collisions between the DNA polymerase and RNA polymerases transcribing these genes (3). The positioning of such genes close to *oriC* is conserved and more so in fast-growing bacteria (4, 5). Repositioning of such genes away from *oriC* or on the lagging strand can be detrimental to fitness, especially under nutrient-rich conditions (6–8).

Can the *oriC-*DnaA dependent mechanism of replication initiation in bacteria be dispensed with? Though DnaA is highly conserved across bacteria, it cannot be detected by sequence homology in a few bacteria (see Table S1 in the supplemental material). Mitochondria are not known to use *oriC-*DnaA-based DNA replication initiation (9, 10). In E. coli, the realization that replication initiation by DnaA is sensitive to inhibition of translation resulted in the discovery of non-*oriC*, non-DnaA-dependent stable DNA replication (SDR) (11).

10.1128/mSphere.00939-19.5 TABLE S1 List of eubacterial strains which did not have a *dnaA* homologue detected in their genomes (out of 5,976 organisms). Download Table S1, PDF file, 0.02 MB. Copyright © 2020 Veetil et al. 2020 Veetil et al. This content is distributed under the terms of the Creative Commons Attribution 4.0 International license.

Multiple broad types of SDR—each with its own set of genetic requirements—have been described. Inducible SDR (iSDR) requires the SOS DNA damage response (11, 12). Constitutive SDR (cSDR) is activated by processes that stabilize RNA-DNA hybrids or R-loops (11), such as the inactivation of (i) RnhA, the RNA-DNA hybrid nuclease RNase HI (13), and that of (ii) the topoisomerase I TopA, which results in hypernegative supercoiling and elevated occurrence of RNA-DNA hybrids (14) Excessive R-loops have also been proposed to occur in strains defective for Rho-dependent transcription termination (15–18), though to our knowledge Rho-dependent transcription termination has not been explicitly associated with cSDR. Inactivation of RecG, a helicase for RNA-DNA hybrids with roles in DNA recombination, can also activate SDR (19–23). Very recently, Raghunathan et al. demonstrated the role of the DNA methylase Dam in suppressing aberrant *oriC*-independent chromosomal replication and showed that the deficiency of this protein conferred SDR and that this mechanism is resistant to RNase HI overexpression (24). We note here that DNA replication by SDR is under normal conditions suboptimal relative to canonical DNA replication. At least one report has described nSDR, a non-*oriC*, non-DnaA-dependent mechanism of chromosome replication employed by E. coli cells transiently during the stationary phase (25).

In this paper, we focus on *ΔrnhA*-induced cSDR in *ΔdnaA* mutants of E. coli K-12. An important question in cSDR is where does DNA replication initiate and what consequence does this have on chromosome organization? The Kogoma group, employing traditional marker frequency analysis (MFA), had identified five “*oriK*” loci at which replication might initiate (26). MFA uses the argument that origin-proximal loci have a higher copy number than the rest of the chromosome in growing cells, even if they are not synchronized, to identify potential origins. Recently, Maduike et al. (27) used a deep-sequencing-based high-resolution version of MFA to identify potential *oriK* sites, which were proximal to those identified by Kogoma’s group. The strongest signal in the Maduike et al. study mapped within the terminus of replication (27). Nishitani and colleagues cloned and screened for fragments of the E. coli chromosome with potential for autonomous self-replication and thereby identified a cluster of fragments again from within the terminus (28). However, both Maduike et al. and Nishitani et al. appear to agree that the terminus sites identified in their studies are not bona fide *oriK* sites (27, 28). In the Maduike et al. study, these terminus signals disappeared in a *Δtus* background in which replication forks trapped within the terminus are released. The authors concluded that the terminus signal may represent trapping of forks originating from initiation sites elsewhere on the chromosome (27). Some of the *ter* sites identified by the Horiuchi group lost their activities in the *Δtus* background, but others did not. The Horiuchi group argued that increased copy number of fragments from the terminus can be attributed to homologous-recombination-based events and not autonomous replication (28). Gowrishankar has synthesized these arguments (29) and, in conjunction with his lab’s finding that RNA-DNA hybrids can occur throughout the chromosome (30), presented the case that cSDR can initiate anywhere on the chromosome; individual cells can initiate replication at different sites, thus generating population-level heterogeneity, and these can well explain the prominent MFA signal within the terminus. In a recent paper, Brochu et al. argued that *ΔtopA-topB* (more so than *ΔtopA-rnhA*) cSDR cells show a strong copy number peak within the terminus suggesting an *oriK* site here, but they did not evaluate it in a *Δtus* background (31). These authors, however, observed that the *ter* peak is maintained in a strain with a large inversion around the *ter*, arguing against this peak being merely a consequence of replication fork trapping events.

Here, we attempt to answer the question of the existence of preferred *oriK* sites by taking the position that peak identification in high-resolution MFA studies of cSDR is complicated by the slow-growth phenotype of the parent strain, which results in weak origin-to-terminus-copy-number gradients. We address this using laboratory evolution experiments, generating suppressors that can generate strong copy number gradients even under the cSDR regime, while also identifying a principle underlying the suppression of the slow-growth phenotype of cSDR.

## RESULTS

### Next-generation-sequencing (NGS)-based MFA of *ΔrnhA-ΔdnaA* strain of E. coli K-12.

The gene *rnhA* encodes the RNase HI nuclease that removes RNA-DNA hybrids. The *ΔrnhA* mutant displays cSDR and therefore suppresses the lethality of *ΔdnaA* and *ΔoriC* mutants (13). We obtained an *ΔrnhA* single deletion mutant by homologous recombination and obtained an *ΔrnhA-ΔdnaA-*pHYD2388 (*dnaA*^+^ *lacZ*^+^) mutant of E. coli K-12 (MG1655) from J. Gowrishankar’s lab (24). To obtain the *ΔrnhA-ΔdnaA* strain, we plated overnight cultures of *ΔrnhA-ΔdnaA-*pHYD2388 (*dnaA*^+^ *lacZ*^+^) on X-Gal (5-bromo-4-chloro-3-indolyl-β-d-galactopyranoside) agar plates. Spontaneous loss of the *dnaA*^+^ pHYD2388 plasmid produced white colonies (*dnaA lacZ* negative), which we selected and propagated as the *ΔrnhA-ΔdnaA* strain. *ΔrnhA* and *ΔrnhA-ΔdnaA* strains showed growth characteristics consistent with prior literature (see Fig. S1 in the supplemental material).

10.1128/mSphere.00939-19.1 FIG S1 *ΔrnhA-ΔdnaA* strain shows reduced growth in LB medium. (A) Growth curves of *rnhA*^+^ *dnaA*^+^, *ΔrnhA*, and *ΔrnhA-ΔdnaA* strains in LB at 37°C and 200 rpm. The *x* axis indicates time, and the *y* axis indicates log_2_ OD_600_. (B and C) Box plots for lag time and growth rate followed by each strain, respectively. The *ΔrnhA-ΔdnaA* strain shows reduced growth rate and extended lag phase compared to the *rnhA*^+^ *dnaA*^+^ strain (*P* ⋘ −1, Wilcoxon test, one-tailed). (D) Spotting assay for *rnhA*^+^ *dnaA*^+^, *ΔrnhA*, and *ΔrnhA-ΔdnaA* strains using different dilutions of cultures (left to right, 10^−3^, 10^−4^, 10^−5^, and 10^−6^) in Luria agar plates incubated at 37°C. Download FIG S1, EPS file, 0.3 MB. Copyright © 2020 Veetil et al. 2020 Veetil et al. This content is distributed under the terms of the Creative Commons Attribution 4.0 International license.

In the rest of this work, we use “*ori*” as an umbrella term, when required, to refer to all sites at which replication initiates: this may include *oriC* itself or *oriK* sites at which cSDR initiates. The terminus is a more complex sequence with multiple, directional replication termination motifs at which the Tus protein traps moving replication forks; we use the generic term “*ter*” to refer to the locus bounded by these termination motifs.

**FIG 1:**
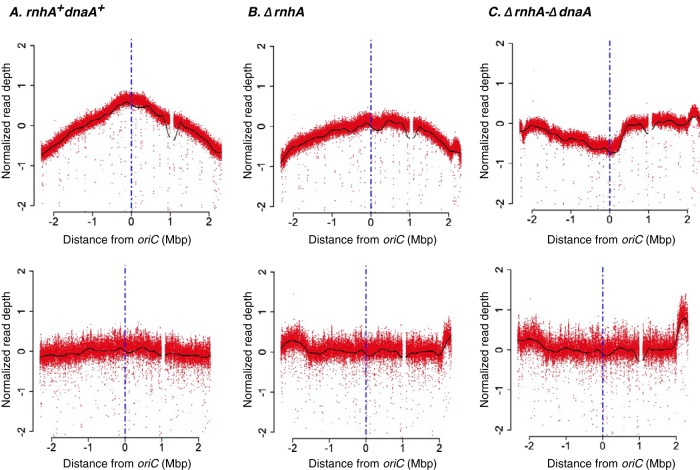
Deep-sequencing-based MFA plots for *rnhA^+^ dnaA^+^*, *ΔrnhA*, and *ΔrnhA-ΔdnaA* strains. The upper panels show the MFA plots for *rnhA*^+^ *dnaA*^+^ (A), *ΔrnhA* (B), and *ΔrnhA-ΔdnaA* (C) strains at the exponential phase of growth, and the lower panels show the same for the stationary phase. The *x* axis represents the distance of a locus either side of *oriC* (in Mbp), with *oriC* itself being the center (blue vertical line). The *y* axis represents the log_2_ values of frequency of reads divided by the mode of the distribution of read counts (see Materials and Methods).

We isolated genomic DNA from *rnhA*^+^ *dnaA*^+^, *ΔrnhA*, and *ΔrnhA-ΔdnaA* strains of E. coli grown to exponential phase—corresponding to the culture’s highest growth rate—in LB. We sequenced the DNA libraries prepared from these samples to an average coverage of ∼200× on the Illumina platform. As controls, we sequenced DNA isolated from stationary-phase populations. For the *rnhA*^+^ *dnaA*^+^ strain, we observed a copy number gradient decreasing from *oriC* toward *ter*, symmetrically on either side of *oriC*, such that the number of reads mapping around *oriC* was 2.48-fold higher than that around *ter* (Fig. 1A). The corresponding plot for stationary-phase cells was relatively flat (Fig. 1A, lower panel).

The *ΔrnhA* mutant, in which both *oriC-*DnaA-dependent replication initiation and cSDR are active, also showed a fairly steep gradient (Fig. 1B). In line with its slow growth, the *ΔrnhA-ΔdnaA* strain showed a flat curve with a few peaks which are candidates for *oriK* sites (Fig. 1C). The strongest peaks were those present within the *ter*, which has been rejected previously (27, 28) as arising as a consequence of trapping of replication forks initiating elsewhere on the chromosome, and a second site around 0.5 to 0.6 Mb clockwise of *oriC*, which we call *oriK45* for its being located at approximately 4.5 Mb into the genome sequence of E. coli K-12 MG1655. In large part, the patterns observed were consistent with those observed by Maduike et al. (27) and Dimude et al. (23) (Fig. 1C and Table 1).

**TABLE 1:** Genomic coordinates mentioned for reference 27 normalized to the respective positions of peaks in E. coli K-12 MG1655 genome version NC_000913.3

Strain	Identified peak (genome coordinates in bp)^*a*^						
	Pos1	Pos2	Pos3	Pos4	Pos5	Pos6	Pos7
Δ*rnhA*-Δ*dnaA*	531400	1449000	1969800	2988600		3699200	4546800
Δ*rnhA*-Δ*dnaA* (27)	790277	1481276	1869776		3228978		4538577
Δ*rnhA*		1453600					4379400
Δ*rnhA* (27)		1471624					4253823

a The *oriC* peaks, when present, are not included in this table.

Whether *oriK45* is a genuine replication initiation site, whether it is indeed a “preferred” site, and whether other minor peaks around the chromosome can represent substantial *oriK* sites remain complicated to answer with the present data set. This is at least in part because of the slow-growth phenotype of the mutant, which ensures that there is hardly any *ori*-*ter* copy number gradient even during periods of its highest growth rate.

### Laboratory evolution experiments of *ΔrnhA-ΔdnaA* strain.

**FIG 2:**
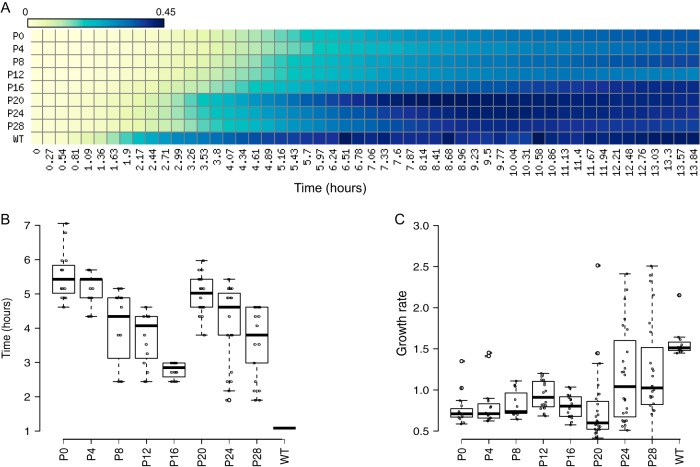
Growth characteristics of evolved mutants. (A) Heat map representing growth of an independently evolved population of *ΔrnhA-ΔdnaA* cells from passage 0 (P0) to passage 28 (P28) based on OD measurements. The *x* axis shows time in hours, the *y* axis shows the number of passages, and the colors represent mean OD values as indicated in the color bar. Similar growth characteristics were observed for other evolution lines. (B and C) Box plots for lag time (B) and growth rate (C) followed by all independent populations. The passage 28 population shows a significantly greater growth rate than that of parental (P0) strains (*P* ⋘ 0.001, Wilcoxon test, one tailed). WT, wild type (*rnhA*^+^ *dnaA*^+^).

To obtain cSDR strains that grow fast and therefore display strong *ori*-*ter* gradients, we performed laboratory evolution experiments in which the *ΔrnhA-ΔdnaA* strain was iteratively diluted into fresh LB and grown to saturation. We used eight independent lines, each derived from a single *ΔrnhA-ΔdnaA* colony subjected to 36 rounds of dilution and growth, corresponding to an estimated 288 generations. Over time, the growth of the population substantially improved (Fig. 2).

We plated aliquots of the culture after each day and noticed the presence of colonies that were visibly larger than those of the parent *ΔrnhA-ΔdnaA* strain. We randomly picked 54 colonies of various sizes—sampling across 3 independently evolved populations and 5 time points and including the 0-day populations—and subjected their genomic DNA to Illumina sequencing. Similarly to our sequencing runs with the parent *ΔrnhA-ΔdnaA* strains, we sequenced DNA isolated from mid-exponential-phase cells. Stationary-phase DNA sequencing was performed for a select few colonies based on genotypes identified from exponential-phase DNA sequencing.

For all these strains, we calculated the ratio between the maxima and the minima of the mid-exponential-phase copy number graphs (see Materials and Methods) and found that this ratio ranged between 0.86 and 2.8 (Table S2). At the lower end, a few colonies showed gradients not too different from the *ΔrnhA-ΔdnaA* parent. The steepest gradients approached, but rarely matched, that of the *rnhA*^+^ *dnaA*^+^ strain.

10.1128/mSphere.00939-19.6 TABLE S2 *ori*-to-*ter* ratios for all strains calculated from MFA plots. To read the sample identifiers, the first number represents the evolution lane (1, 5, and 8), followed by D and the number which represents day of evolution (0, 4, 8, 12, and 15), and the last number represents the number of colonies selected from that population. For example, 5D0_3 means third colony selected from day 0 of evolution of evolution lane number 5. Download Table S2, PDF file, 0.03 MB. Copyright © 2020 Veetil et al. 2020 Veetil et al. This content is distributed under the terms of the Creative Commons Attribution 4.0 International license.

### Large inversions around *oriC* suppress the growth defect of *ΔrnhA-ΔdnaA* mutant.

**FIG 3:**
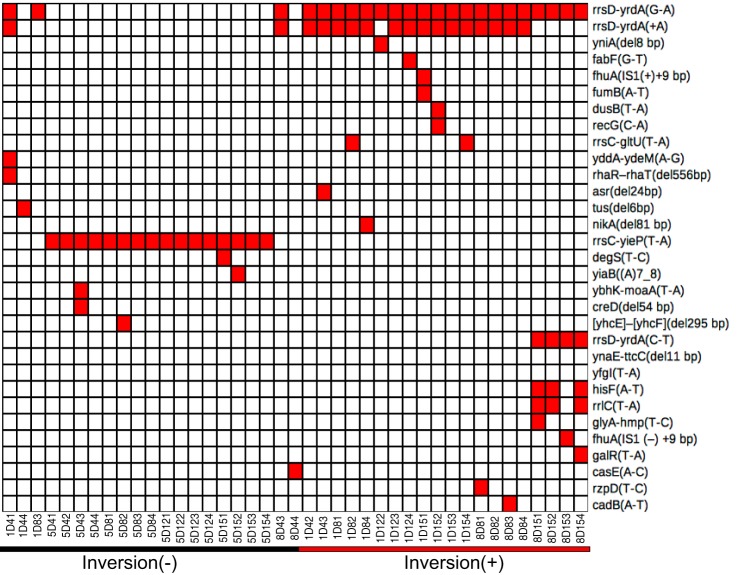
Unique mutations in suppressor mutants. Heat map representing unique mutations (100% frequency) in all independent colonies sequenced generated using Matrix2png. Color represents the presence of a mutation in the respective gene shown on the *y* axis. The *x* axis represents sample identifiers of suppressor mutants evolved from three independent populations. Presence and absence of chromosomal inversions are represented using red and black lines, respectively. Mutations in pseudogenes and those also found in parental strains are listed in Fig. S2.

We next used these sequencing data to identify mutations—both point variations including indels and structural variations such as large amplifications, deletions, and inversions. Large amplifications and deletions can be identified by sharp local increases or decreases, respectively, in copy number. Inversions can be detected as local flips in copy number plots of exponential-phase genomic DNA sequencing data with clear *ori*-*ter* gradients (32). We found several point mutations in the evolved clones not present in the *ΔrnhA-ΔdnaA* parent (Fig. 3 and Fig. S2). Approximately 90% of colonies carried a mutation upstream of one of two rRNA operons, *rrnD* and *rrnC.* One clone carried an in-frame deletion mutation in *tus* (Δ6 bp [1684458 to 1684463]), which translates to a QSL-L variation in the amino acid sequence. We did not find any amplification, and the only deletion that was apparent in the data was an ∼97-kb ([*mmuP*] to [*mhpD*]) deletion around the *lac* locus, which is part of the genotype of the *rnhA*^+^ *dnaA*^+^ founder strain used in this study (24).

10.1128/mSphere.00939-19.2 FIG S2 Plot represents mutational matrices representing pseudogene mutations and common mutations (which are present in parental strains) for independent colonies sequenced. Red indicates the presence of the mutation in the gene shown on the *y* axis. The *x* axis shows the sample identifiers of suppressor mutants and parental strains evolved from three independent populations. Download FIG S2, EPS file, 0.09 MB. Copyright © 2020 Veetil et al. 2020 Veetil et al. This content is distributed under the terms of the Creative Commons Attribution 4.0 International license.

**FIG 4:**
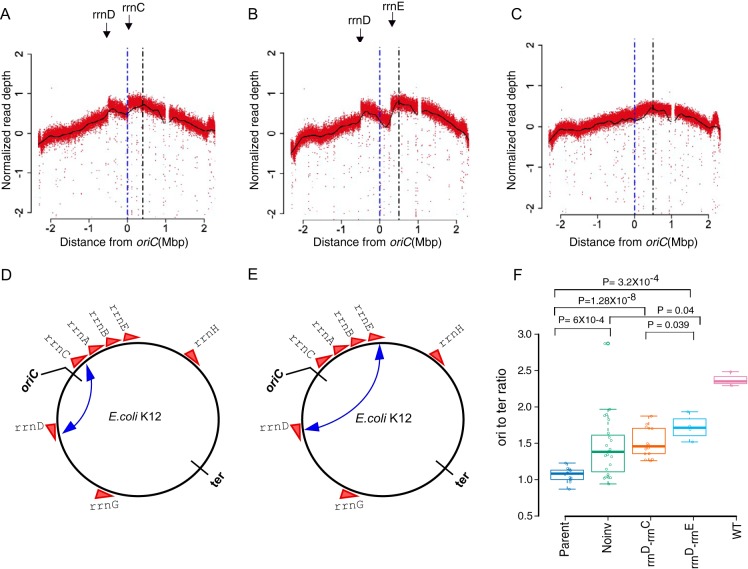
Deep-sequencing-based MFA plots for suppressor mutants. (A to C) MFA plots for *ΔrnhA-ΔdnaA inv^rrnD-rrnC^* (A), *ΔrnhA-ΔdnaAinv^rrnD-rrnE^* (B), and *ΔrnhA-ΔdnaA Noinv* (C) sequenced at the exponential phase of growth. The dashed blue line represents the *oriC* position, and the black line represents the position at maxima of LOESS fit value. Plots in panels A and B show the presence of different chromosomal inversions flanked by *rrn* operons (mentioned above). (D and E) The position of the inversion on the chromosome schematically represented, corresponding to panels A and B, respectively. (F) Box plot representing *ori*-to-*ter* ratio differences in different populations of evolved clones compared to wild-type E. coli. *x* axis labels: Parent, parent-*ΔrnhA-ΔdnaA* strain passage 0 clones; Noinv, suppressor mutants which do not show the presence of chromosomal inversion; *rrnD-rrnC*, clones which show the presence of a chromosomal inversion from *rrnD-rrnC* (*ΔrnhA-ΔdnaA inv^rrnD-rrnC^*); *rrnD-rrnE*, clones which show the presence of a chromosomal inversion from *rrnD-rrnE* (*ΔrnhA-ΔdnaA inv^rrnD-rrnE^*); WT, wild type (*rnhA*^+^ *dnaA*^+^).

**FIG 5:**
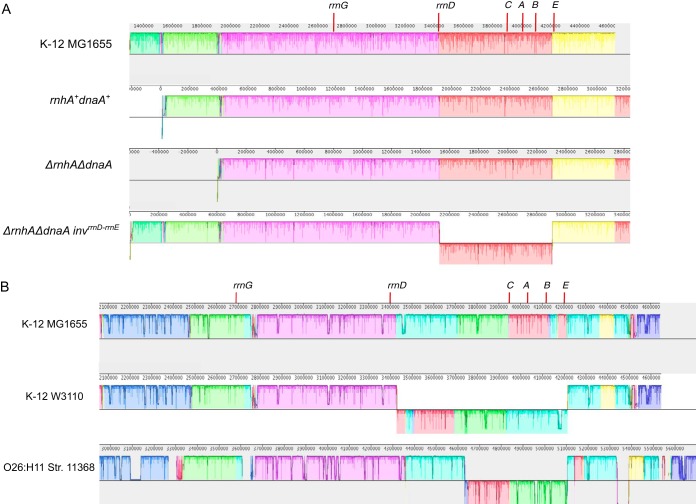
(A) Plot showing sequence alignment of *de novo*-assembled contigs obtained from Nanopore sequencing for *rnhA*^+^ *dnaA*^+^, *ΔrnhA-ΔdnaA*, and *ΔrnhA-ΔdnaA inv^rrnD-rrnE^* strains with respect to E. coli K-12 MG1655 genome. The strand shift of the red color bar for the *ΔrnhA-ΔdnaA inv^rrnD-rrnE^* strain shows the presence of an ∼0.8-Mb chromosomal inversion. The position of the *rrn* operons near the *ori* region is marked. (B) Figure representing sequence alignment of a selected region of the chromosome for three E. coli strains. E. coli K-12 W3110 and E. coli O26:H11 strain 11368 show the presence of a chromosomal inversion around the *oriC* region compared to the reference E. coli K-12 strain MG1655. The strand shift for the colored bars represents chromosomal inversion. The position of the *rrn* operons near the *ori* region is marked. These images were generated using the software Mauve.

We found inversions around *oriC* in ∼45% of the evolved colonies (Fig. 3 and 4). One end of these inversion was *rrnD*, located 3.42 Mb counterclockwise of *oriC* in the reference genome of E. coli K-12 MG1655. In ∼80% of inversions, the other end was *rrnC* (3.94 Mb), and in the remaining, the second end was *rrnE* (4.2 Mb). The *rrnD-rrnC* inversion (*ΔrnhA-ΔdnaA inv^rrnD-rrnC^*) measured ∼0.5 Mb and the *rrnD-rrnE* (*ΔrnhA-ΔdnaA inv^rrnD-rrnE^*) measured ∼0.8 Mb (Fig. 4). We used long-read nanopore sequencing to assemble the genome of the clone with the longer *rrnD-rrnE* inversion into just one contig *de novo* and confirmed the presence of the inversion (Fig. 5A).

Thus, both inversions would move a set of rRNA operons from clockwise to counterclockwise of *oriC* and move the *rrnD* operon in the opposite direction. Irrespective of the presence of the inversion, all these rRNA operons would continue to lie on the leading strand of canonical replication from *oriC*. That the fitness cost of these inversions would be minimal under conditions of normal DNA replication is also suggested by the fact that inversions bounded by at least one *oriC*-proximal rRNA operon are found in 37 other E. coli genomes (out of 675 considered), including another strain of E. coli K-12 (W3110) (33) (Fig. 5B and Table S3). Colonies with either inversion in the present study also carried the following mutations upstream of *rrnD*: (i) G-A (position 3429052) and +A (3429054) or (ii) C-T (3429055) (Fig. 3).

10.1128/mSphere.00939-19.7 TABLE S3 List of E. coli strains in which the presence of a chromosomal inversion around *oriC* is observed. Download Table S3, PDF file, 0.02 MB. Copyright © 2020 Veetil et al. 2020 Veetil et al. This content is distributed under the terms of the Creative Commons Attribution 4.0 International license.

We then compared the maximum-minimum ratios in the copy number plots of clones (not considering the peak within *ter*) with the two types of inversions and those without. For this analysis, we grouped all colonies without an inversion together, fully aware that this is a genetically heterogeneous group. Clones with the longer *rrnE-rrnD* inversion showed significantly higher maximum-minimum ratios than those with the shorter *rrnC-rrnD* inversion (*P* = 0.039, Wilcoxon test, one-tailed) (Fig. 4F). Therefore, the longer inversion appears to be a better suppressor of the growth defect of cSDR than the shorter inversion. Many clones without the inversions, including the one with the Δ6-bp in frame deletion mutation in Tus, showed substantially smaller maximum-minimum ratios, though a few colonies did show higher values.

### *oriK45* as a preferred initiation site for cSDR in suppressors.

**FIG 6:**
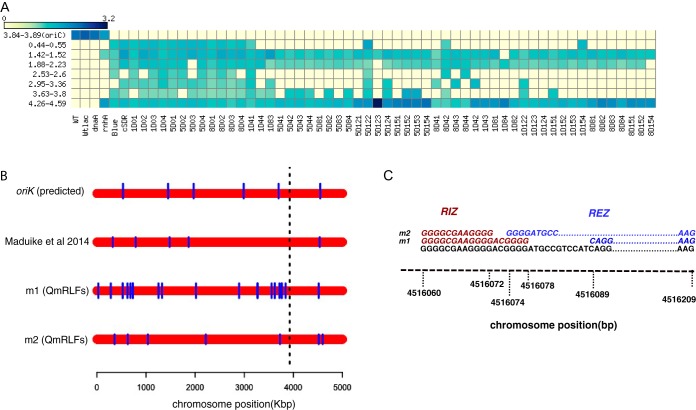
*oriK45* as a preferred initiation site for cSDR in suppressor mutants. (A) Heat map showing predicted *oriK* position ranges from marker frequency analysis across evolved strains. The *y* axis represents the chromosomal positions of predicted *oriK* ranges in Mbp. Color indicates the ratio of corresponding LOESS smoothed normalized read count to the LOESS minima of the plot at the peak. (B) Plot showing positions of R-loops predicted by m1 and m2 models of QmRLFs on the E. coli chromosome in comparison with positions of predicted *oriK* sites in the *ΔrnhA-ΔdnaA* strain from the work of Maduike et al. (27) and the *oriK* sites for the same strain mentioned in this study. Each red bar represents the bacterial chromosome, on which the R-loop positions are marked in blue lines. (C) Sequence motif of R-loop initiation zone (RIZ) and R-loop elongation zone predicted by m1 and m2 models of QmRLFs around the 4.51-Mb region of the E. coli K-12 MG1655 chromosome.

We identified the locations of the maxima of the copy number curve for the suppressors, while ignoring the *ter* peak. We noticed that these, across all cSDR strains used in this study, mapped to ∼4.3 Mb to 4.6 Mb clockwise of *oriC*, in proximity to *oriK45* (Fig. 6A). Consistent with this, all suppressors showed a copy number peak at *oriK45* (Fig. 6A and Table S4). This suggests that *oriK45* is a predominant site of cSDR initiation in *all* suppressors identified here.

10.1128/mSphere.00939-19.8 TABLE S4 Chromosomal position of predicted *oriK* peaks from MFA plots. The value represents LOESS fit value for the maximum position of the peak identified. Download Table S4, PDF file, 0.03 MB. Copyright © 2020 Veetil et al. 2020 Veetil et al. This content is distributed under the terms of the Creative Commons Attribution 4.0 International license.

In the strongest suppressors, we observed a strong copy number gradient peaking at *oriK45* and declining toward *ter* (Fig. 4A and B) (DNA copy number curves for all evolved mutants are available at https://doi.org/10.6084/m9.figshare.11800299). The peak in *ter* was computationally detected in all suppressors. However, this peak was weak in two of the suppressors. One of these contained a 6-bp in-frame deletion mutation in *tus* (Fig. 3, sample ID 1D4_4) and displayed a copy number pattern similar to that observed for *Δtus* by Maduike et al., (27), indicating that the mutation observed here causes loss of function. This strain did show a slight copy number peak at *oriK45*, but its being a relatively weak suppressor does not permit a more confident assignment.

### *oriK45* is proximal to predicted R-loop-forming sites.

We asked whether *oriK45* is proximal to regions with high propensities to form RNA-DNA hybrids. We used a computational technique that searches for two G-rich patterns on a given DNA sequence to identify loci that have the propensity to form RNA-DNA hybrids (34, 35). This method predicted ∼30 R-loop-favoring sites, showing homology to at least one of the two RNA-DNA hybrid-forming sequence patterns, across the E. coli chromosome (Fig. 6B). Eight of the 11 copy number bumps described by us or by Maduike et al. (27) for the *ΔrnhA-ΔdnaA* strain were within 200 kb of at least one of the predicted sites. This is statistically significant compared to random assignment of genome coordinates to experimentally predicted copy number peaks (*P = *10^−5^, Z-score, permutation test across 1,000 repetitions, one-tailed). However, only one site showed homology to both RNA-DNA hybrid-forming sequence patterns; this site is at 4.51 Mb (Fig. 6C), within the range defined by *oriK45*.

Leela et al. (30) had identified bisulfite-sensitive regions of the E. coli chromosome and defined these as preformed R-loops. However, we did not find any statistically significant overlap of these sites with *oriK45*. Nevertheless, we found two clusters of highly bisulfite-sensitive genes in the *oriK45* region and also observed that the R-loop-forming sequence mentioned above was also highly bisulfite sensitive.

Nishitani et al., while screening for genomic DNA fragments capable of autonomous replication, described a site called *hotH*, which is at 4.55 to 4.56 Mb (28). However, to our knowledge, these authors did not report further exploration of the *hotH* site and focused instead on the characterization of the cluster of fragments from within *ter*. Among the transposon insertions found to affect replication of *ΔtopA*-mediated cSDR is an insertion within *fimD*, which is again in the region defined by *oriK45* (36).

### cSDR from *oriK45* has a pleiotropic effect on gene expression.

What are the effects of cSDR on gene expression—as measured by global patterns along the length of the chromosome and signatures on pathways related to DNA replication, repair, and transcription? To what extent does the suppression of growth defects of cSDR by the inversion around *oriC* reverse these effects? Toward answering these questions, we performed exponential-phase transcriptome analysis of *rnhA*^+^ *dnaA*^+^, *ΔrnhA*, *ΔrnhA-ΔdnaA*, *ΔrnhA-ΔdnaA inv^rrnD-rrnC^*, and *ΔrnhA-ΔdnaA inv^rrnD-rrnE^* strains using transcriptome sequencing (RNA-seq).

Both *ΔrnhA* and *ΔrnhA-ΔdnaA* induced large changes in gene expression compared to *rnhA*^+^ *dnaA*^+^. Six hundred genes were upregulated and 543 were downregulated by a log(base 2) fold change of 1.5 or above in the *ΔrnhA-ΔdnaA* strain. The corresponding numbers for *ΔrnhA* are 472 and 360, respectively. Nearly 75% of all genes induced in the *ΔrnhA* strain were also induced in the *ΔrnhA-ΔdnaA* strain, the proportion for downregulated genes being ∼80%. Despite the overlap in these gene lists, the magnitude of differential expression was in general less in the Δ*rnhA* than in the *ΔrnhA-ΔdnaA* strain (*P < *10^−10^, paired Wilcoxon test comparing magnitudes of differential expression). Functional classification of differentially expressed genes using Clusters of Orthologous Groups (COGs) showed an enriched upregulation (*P < *0.01, Fisher’s exact test) of various classes of genes including replication, recombination, and repair genes; ion transport and metabolic genes; and translation genes (Table S5). On the other hand, cell motility, energy production and conversion, carbohydrate transport, and metabolic genes showed a significant downregulation in the *ΔrnhA-ΔdnaA* strain.

10.1128/mSphere.00939-19.9 TABLE S5 Functional classification of genes based on COG categories. Enrichment of a functional category is marked in yellow. Download Table S5, PDF file, 0.04 MB. Copyright © 2020 Veetil et al. 2020 Veetil et al. This content is distributed under the terms of the Creative Commons Attribution 4.0 International license.

Genes encoding several members of the SOS response, including the cell division inhibitor SulA, error-prone polymerases DinB and UmuC, and RuvB and RuvC, are upregulated in both *ΔrnhA* and *ΔrnhA-ΔdnaA* strains. *dinF*, the SOS-inducible gene that also confers protection against oxidative stress, was induced in both the mutants. Other signatures for an oxidative stress response included the induction of *sufB–E*, whose protein products are involved in iron-sulfur cluster biogenesis under oxidative stress (37). Very few members of the general stress response (∼6%; underrepresented compared to sigma-70 targets, *P = *4 × 10^−6^, Fisher’s exact test), defined as targets of sigma-38 (RpoS), were induced.

We also observe an upregulation of *holB* and *holD*, encoding the delta-prime and the epsilon subunits, respectively, of the replicative DNA polymerase III. This might in part be consistent with the SOS response, in light of the evidence that induction of SOS-responsive DNA polymerases can be lethal in a genetic background that is defective for HolD (38). The gene *topA*, encoding topoisomerase, which can decrease R-loop formation, presumably through its DNA-relaxing activity, is also upregulated.

We observe that several genes encoding components of the ribosome are upregulated in the inversion mutants. At least three DEAD box RNA helicase genes (*rhlE*, *dbpA*, and *srmB*) that are involved in ribosome assembly are also upregulated. Finally, *rapA*, the gene encoding the RNA polymerase recycling factor ATPase, which is required for reloading stalled RNA polymerase, is upregulated.

### Gene expression changes show limited but significant correlation with DNA copy number changes.

**FIG 7:**
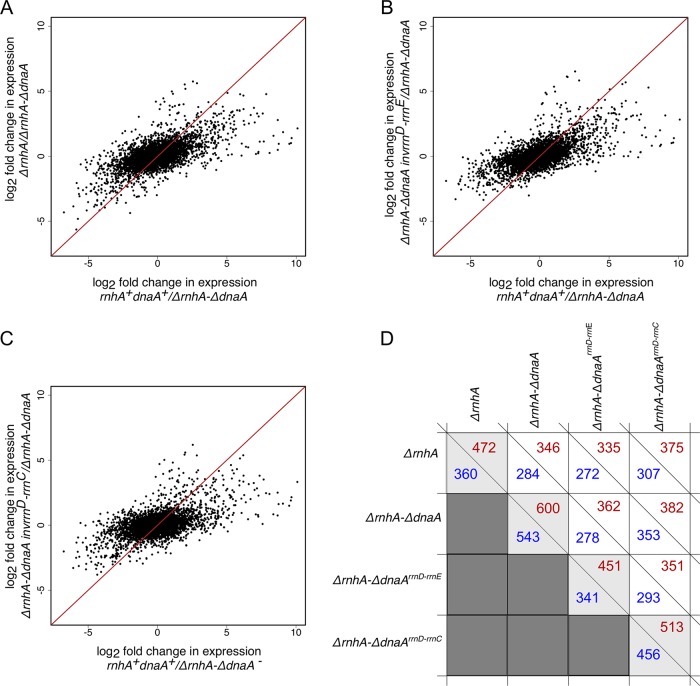
Scatter plots representing correlation of log_2_ fold change in gene expression for different conditions, compared to the *ΔrnhA-ΔdnaA* strain. (A) *ΔrnhA* versus *rnhA*^+^ *dnaA*^+^ strain. (B) *ΔrnhA-ΔdnaA inv^rrnD-rrnE^* versus *rnhA*^+^ *dnaA*^+^ strain. (C) *ΔrnhA-ΔdnaA inv^rrnD-rrnC^* versus *rnhA*^+^ *dnaA*^+^ strain. The Pearson correlation values for panels A to C are 0.638, 0.639, and 0.553, respectively. (D) Plot representing the number of upregulated (red) and downregulated (blue) genes for all strains compared with the *rnhA*^+^ *dnaA*^+^ strain.

Overall, there is a gradient—decreasing from *oriC* toward *ter*—in the fold change in gene expression between *rnhA*^+^ *dnaA*^+^ and *ΔrnhA-ΔdnaA* strains. In other words, genes that are proximal to *oriC* (and *oriK45*) are more strongly downregulated in the *ΔrnhA-ΔdnaA* strain compared to the *rnhA*^+^ *dnaA*^+^ strain (Fig. S3). At this level, the fold change in the *rnhA*^+^ *dnaA*^+^ strain, in relation to the *ΔrnhA-ΔdnaA* strain, shows strong similarity to that in *ΔrnhA* and *ΔrnhA-ΔdnaA inv^rrnD-rrnE^* strains (Pearson correlation coefficient = 0.64 for both comparisons) and slightly less similarity to the *ΔrnhA-ΔdnaA inv^rrnD-rrnC^* strain (Pearson correlation coefficient = 0.55) (Fig. 7). These indicate that a portion of the gene expression change in the *ΔrnhA-ΔdnaA* strain relative to the *rnhA*^+^ *dnaA*^+^ strain is reversed by the longer inversion *ΔrnhA-ΔdnaA inv^rrnD-rrnE^* and probably less so by the shorter inversion *ΔrnhA-ΔdnaA inv^rrnD-rrnC^*. Nevertheless, the magnitude of the difference in gene expression between *rnhA*^+^ *dnaA*^+^ and *ΔrnhA-ΔdnaA* strains is higher than that between the suppressors and the *ΔrnhA-ΔdnaA* strain (*P < *10^−10^, paired Wilcoxon test comparing magnitudes of differential expression).

10.1128/mSphere.00939-19.3 FIG S3 Plots showing the trend followed by the smoothened log_2_ fold change values for all genes in comparison with *ΔrnhA-ΔdnaA* strain for gene expression and DNA copy number. (A) *rnhA*^+^ *dnaA*^+^/*ΔrnhA-ΔdnaA* strain. (B) *ΔrnhA/ΔrnhA-ΔdnaA* strain. (C) *ΔrnhA-ΔdnaA inv^rrnD-rrnE^/ΔrnhA-dnaA* strain. (D) *ΔrnhA-ΔdnaA inv^rrnD-rrnC^/ΔrnhA-ΔdnaA* strain. The *x* axis represents positions centered around *oriC*, and the *y* axis represents LOESS fit values of log_2_ fold change. Red lines represent gene expression, and black lines represent DNA copy number for the same strain. Download FIG S3, EPS file, 0.2 MB. Copyright © 2020 Veetil et al. 2020 Veetil et al. This content is distributed under the terms of the Creative Commons Attribution 4.0 International license.

A small but statistically significant portion of the difference in gene expression can be explained by differences in DNA copy number—a consequence of differences in maximal growth rates—as measured by NGS of matched exponential-phase genomic DNA samples (Pearson correlation coefficient ∼0.2, *P < *10^−10^). These correlations between DNA copy number and RNA-seq-based gene expression fold changes increase to over 0.75 in all comparisons when gene expression data are smoothed by locally estimated scatterplot smoothing (LOESS), which averages out local variation in expression levels.

The movement of the origin of replication to *oriK45*, and the large inversion, might affect the macrodomain structure of the chromosome (39), as well as supercoil gradients (40). *oriK45* would be located at the right extreme of the *ori* macrodomain. The left end of the larger inversion is within a nonstructured region of the chromosome, whereas the right end is within the *ori* macrodomain, and such an inversion could have consequences for cell physiology as well as gene expression (41). What the precise effect of these chromosome structure parameters is on the transcriptional profile, in the absence of chromosome conformation data under cSDR, is not clear at the moment.

Therefore, overall gene expression changes along the chromosome are weakly correlated with the distance of a gene from *oriC* (and *oriK45*) and changes in DNA copy number. Gene expression changes that occur in the *ΔrnhA-ΔdnaA* strain relative to the *rnhA*^+^ *dnaA*^+^ strain are partly compensated by inversion-containing suppressors.

### The inversions reduce replication-transcription conflicts at rRNA loci but not at essential mRNA genes.

To understand the impact of inversions on transcription-replication collisions, we calculated a fractional score for the occurrence of head-on collisions for genes on the lagging strand with respect to replication from *oriC* or *oriK45* using RNA sequencing data (see Materials and Methods). This score was lowest at 0.31 for the *rnhA*^+^ *dnaA*^+^ strain. This increased to 0.67 in the *ΔrnhA-ΔdnaA* strain but was reduced to 0.39 in the suppressor *ΔrnhA-ΔdnaA inv^rrnD-rrnE^* strain (Table 2). This effect was the strongest when only rRNA genes (5S rRNA, which is not depleted as part of the RNA preparation experiment) were considered. Despite the large decrease in replication-transcription conflict in the inversion-containing suppressors, the activation of the SOS response in cSDR is not reversed, even at a quantitative level; this requires further investigation.

**TABLE 2:** Probability of head-on collisions predicted for the 3.3-Mbp to 4.25-Mbp region of the chromosome^*a*^

Gene group and strain	Head-on collision rate
All genes	
*rnhA*^+^ *dnaA*^+^ strain	0.31
Δ*rnhA*-Δ*dnaA* strain	0.67
Δ*rnhA*-Δ*dnaA^rrnD-rrnE^* strain	0.39
Ribosomal genes	
*rnhA*^+^ *dnaA*^+^ strain	0.22
Δ*rnhA*-Δ*dnaA* strain	0.84
Δ*rnhA*-Δ*dnaA^rrnD-rrnE^* strain	0.24

a Probability of head-on collisions [*P*(HO)] was predicted for the 3.3-Mbp to 4.25-Mbp region of the chromosome which includes the inverted region of the Δ*rnhA*-Δ*dnaA^rrnD-rrnE^* strain. The values were calculated by taking the ratio of the sum of RNA coverage values of all genes on the lagging strand with respect to the single predominant *ori* position to the total RNA coverage for the region [*P*(HO) = sum(lagging-strand RNA coverage)/total RNA coverage]. The analysis was done for different classes of genes separately by using the functional annotations for genes from the NC_000913.3 file (.ptt and .rnt).

Curiously, however, clashes appeared to increase for mRNA genes, including essential genes; it must, however, be noted that the expression levels of mRNA genes would be only a fraction of rRNA levels. Therefore, it appears that any suppression in the growth defect may arise from a reversal of increased replication-transcription conflicts at rRNA loci, notwithstanding any effect on essential or nonessential mRNA genes.

## DISCUSSION

Taken together, our results indicate that under *ΔrnhA-ΔdnaA* cSDR, selection favors preferential replication initiation from *oriK45*, located ∼0.4 to 0.7 Mb clockwise of *oriC*. *oriK45* is a broadly defined region and spans an ∼300-kb region across the samples analyzed here. The precise location of one or more initiation sites within *oriK45* is unknown and may be beyond the capabilities of MFA experiments in unsynchronized populations to determine. The top homology to R-loop-forming sequences is at ∼4.51 Mb; *hotH*, previously shown to be capable of autonomous replication (28), is ∼40 to 50 kb clockwise of the above R-loop-forming sequence; *fimD*, an insertion in which has an effect on *ΔtopA*-mediated cSDR (36), is located between the above two sites and is closer to *hotH*. These three sites, while located in the broad region that defines *oriK45*, do not overlap. There could be multiple discrete initiation sites within *oriK45*, or *oriK45* might encompass a region with diffuse initiation points.

Are there one or more *oriK* sites within *ter*? Though the *ter* peak reported by Maduike et al. and Dimude et al. (23, 27) disappeared in a *tus* mutant (as well as in a mutant carrying a small deletion in *tus* in our study), which accounts for fork trapping, this evidence may not fully eliminate the possibility of a relatively weak *ter oriK*. The absence of a strong *ori-ter* gradient in these slow-growing *tus* mutants may always cause such a peak to be missed. Though we observe that the *ter* peak is retained in our stationary-phase cells, there is still the possibility that there is still some cSDR activity from a *ter*-proximal *oriK* site in these cells. That said, however, *oriK45* appears to be favored by selection, and the fact that this site is located relatively close to the canonical *oriC* may help its cause. One potential future experiment would be an analysis of a strain that combines the inversion-containing suppressors isolated in our study with a *Δtus* mutation. In such a mutant, a strong *oriK* site within *ter* might manifest as an obvious peak but might come with the cost of dramatically upsetting the highly favorable copy number gradient declining from *oriC* toward *ter.*

Replication initiation from *oriK45* would result in head-on collisions with RNA polymerases transcribing four rRNA operons carried between *oriC* and *oriK45*. Such head-on collisions are detrimental at least in part because of DNA topological issues that cause excessive R-loop formation in such conflict sites (42). The predominant suppressor found here would invert the DNA around *oriC* such that these four rRNA operons would now be on the leading strand of replication from *oriK45*. This would, however, place one rRNA operon now on the lagging strand. The promoter of this rRNA operon carried a mutation in the discriminator region in all inversion-carrying suppressor strains. Though we could not find any significant difference in the expression levels of plasmid-borne green fluorescent protein (GFP) cloned downstream of the wild-type *rrnD* promoter and that with the discriminator mutation (see Fig. S4 in the supplemental material), whether this mutation confers a specific ppGpp-dependent effect on gene expression in a cSDR background and whether this affects fitness remain to be understood. Recent evidence shows that certain genes—including determinants of virulence and antibiotic resistance—over the course of evolution might have switched in the reverse direction, from leading to lagging (43). Such genes experience higher rates of nonsynonymous mutations, experiencing positive selection and thereby promoting evolvability. However, this would not apply to highly expressed genes such as the rRNA genes.

10.1128/mSphere.00939-19.4 FIG S4 Plots showing the respective GFP fluorescence intensity measured using fluorescence-activated cell sorting for *pUA139* (orange), *wt_rrnD_IGR_pUA139* (blue), and *mut_rrnD_IGR_pUA139* (red) strains at different time intervals, 0 h (A), 2 h (B), and 5 h (C). Download FIG S4, EPS file, 0.06 MB. Copyright © 2020 Veetil et al. 2020 Veetil et al. This content is distributed under the terms of the Creative Commons Attribution 4.0 International license.

In a previous study, the Sherratt lab placed a second *ori* termed *oriZ* ∼1 Mb clockwise of *oriC*. They reported that replication initiation from *oriZ*, despite *oriZ* being positioned such that it would cause replication-transcription conflicts at rRNA operons, caused few replication or growth defects (44). However, a later attempt by Ivanova and colleagues to create a similar strain revealed a strong growth defect and also showed that mutations that allow the RNA polymerase to bypass conflicts efficiently and those that inactivate *ter* can suppress the growth defect (45). MFA of the Sherratt lab strain by Ivanova et al. indicated the presence of a large inversion, affecting several rRNA operons, which had not been detected by the Sherratt study (44). The inversion reported by Ivanova et al. (45) is similar to that observed in our study, except that the right end reported by the earlier study extends beyond that found by us to a position closer to that of *oriZ*. Thus, Ivanova et al. could conclude that replication-transcription conflicts are key determinants of fitness of E. coli. These findings are consistent with those of Srivatsan et al., who showed that a large *oriC*-proximal inversion can cause growth defects when Bacillus subtilis is grown in rich medium (8). In contrast to these findings, Esnault et al. showed that inversions near *oriC* which would place 1 to 3 rRNA operons on the lagging strand of replication showed little growth defect (41). That the inversion observed in our study contributes to fitness may be ascertained from the fact that the larger inversion produces higher copy-number gradients than the smaller inversion, although both strains carry the *rrnD* promoter mutation. The selective advantage conferred by the inversion also indicates that replication initiates predominantly clockwise of *oriC*, from a position that is also clockwise of the four rRNA operons that are inverted. *oriK45* satisfies these requirements.

Structural variations around *ter* have also been found to exist in E. coli with a second *ori*. Dimude et al. (46) placed a second *ori*, termed *oriX*, counterclockwise of *oriC*. They found that this mutant carried an ∼0.8-Mb inversion spanning the *ter* (46). However, this mutant grew slowly. Since the authors did not isolate an *oriX*^+^ strain without the inversion, they were unable to directly test whether it conferred a selective advantage, even if a small one, on its parent.

Whereas the previous studies by Ivanova et al. and Dimude et al., (45, 46) isolated structural variations while creating the parent strain, we were able to isolate our suppressors only after 4 to 8 days of selection in a laboratory evolution experiment.

Though cSDR may not necessarily be a physiological or natural phenomenon in E. coli, with the possible exception of its manifestation as nSDR in stationary phase, it has been argued that this could be a potential primordial mechanism of DNA replication initiation (11). Further, cSDR can provide the bacterium avenues for the development of resistance to new antibiotics targeting initiation of DNA replication (47, 48).

## MATERIALS AND METHODS

### Strains and medium conditions.

The wild-type (*rnhA*^+^ *dnaA*^+^) strain mentioned in this study is a derivative of a nonpathogenic *E.coli* K-12 MG1655 strain named GJ13519 in reference 30. Gene deletions were performed using the one-step inactivation method described by Datsenko and Wanner (49) or by P1 phage-mediated transduction (50). All experiments were conducted in Luria-Bertani (LB; Hi-Media, India; catalog no. M575-500) broth to select for suppressors at a higher rate than under slow-growing minimal medium conditions used in previous studies of cSDR. Higher growth rates also produce stronger *ori-ter* gradients, which enable better peak identification. Where required, the strains were grown in the presence of antibiotics kanamycin, ampicillin, and trimethoprim at final concentrations of 50, 50, and 10 μg/ml, respectively.

Growth curves were generated in 250-ml flasks or 24-well plates in Luria-Bertani (LB; Hi-Media, India; catalog no. M575-500) broth at 37°C with shaking at 200 rpm. Optical density (OD) measurements were carried out at 600 nm (OD_600_) using s UV-visible spectrophotometer (SP-8001) or a multiwell plate reader (Infinite F200pro; Tecan). Growth rates were calculated using Growthcurver (https://CRAN.R-project.org/package=growthcurver), and all plots were generated using customized R scripts.

### Spotting assay.

The spotting assay was performed for all strains at μ_max_, which corresponds to the maximum growth at the exponential phase of growth. Overnight-grown bacterial cultures were diluted in LB medium to achieve an 0.03 OD and incubated at 37°C and 200 rpm until reaching μ_max_. Serial 10-fold dilutions of cultures were spotted (as 3-μl spots) on LB agar plates. The plates were imaged after 30 h of incubation at 37°C.

### Whole-genome sequencing and DNA copy number analysis.

For genomic DNA extraction, the overnight cultures were inoculated in 50 ml of fresh LB medium to bring the initial optical density (OD) of the culture to 0.03, and the flasks were incubated at 37°C with shaking at 200 rpm. Cells were harvested at μ_max_, and genomic DNA was isolated using the GenElute bacterial genomic DNA kit (NA2120-1KT; Sigma-Aldrich) using the manufacturer’s protocol. Library preparation was carried out using the TruSeq Nano DNA low-throughput library preparation kit (15041757), and paired-end (2 × 100) sequencing of genomic DNA was performed on the Illumina HiSeq 2500 platform. For stationary-phase whole-genome sequencing, the cultures were harvested after 16 h of growth.

The sequencing reads were aligned and mapped to the reference genome (NC_000913.3) using the Burrows-Wheeler aligner (BWA) (51) specifying alignment quality and mapping quality thresholds as 20. Read coverage across the genome was calculated for nonoverlapping windows of 200 nucleotides (nt) each using custom PERL scripts, and the values were normalized by the mode of the distribution across these bins. The normalized values in logarithmic scale (log_2_) were plotted against chromosome coordinates to get measures of DNA copy number from *ori* to *ter.* The coordinates were repositioned in such a way that the numbering starts from the *oriC* position in either direction. LOESS polynomial regression analysis was used for curve fitting.

### Laboratory evolution of cSDR mutant.

The laboratory evolution experiment was carried out for overnight-grown cultures of eight independent *ΔrnhA-ΔdnaA* isolates. Cells were grown in 24-well plates at 37°C, with shaking at 200 rpm, until late exponential phase and diluted by a factor of 1:100 into fresh LB broth. Bacterial populations were stored as 50% glycerol stocks at −80°C before the next subculturing. A contamination check was done for each population using PCR amplification of *rnhA* and *dnaA* genes from isolated genomic DNA samples. Alternative passages were plated on Luria agar plates (10^−6^ and 10^−7^ dilutions), and CFU/ml for each sample was counted during the course of evolution. The number of generations of evolution (*N*) was calculated using the minimum and maximum OD values per passage. The growth characteristics of evolved populations were monitored in 96-well plates at 37°C, 200 rpm, using a plate reader (Tecan; Infinite F200 Pro). Randomly chosen colonies from different passages were selected for whole-genome sequencing.

### Mutation analysis and *ori*-to-*ter* ratio calculation.

Single nucleotide polymorphisms (SNPs) and indels were identified from the genome sequencing data using the breseq (version 0.33.1) (52) pipeline, which uses Bowtie for sequence alignment. A mutational matrix representing presence and absence of mutations was generated from the breseq output file using custom R scripts, and heat maps were generated using Matrix2png (53). Copy number plots for each sample at the maximum growth rate were used to determine *ori*-to-*ter* ratios. The ratio of maximum LOESS fit value (excluding *ter*) to the LOESS fit value of the *dif* site (1,588,800) for each evolved strain was calculated using custom scripts.

### Nanopore sequencing and assembly of genomes.

Genomic DNA isolated using the GenElute bacterial genomic DNA kit (NA2120-1KT; Sigma-Aldrich) was subjected to nanopore sequencing. Sequencing library preparation was carried out with nanopore genomic sequencing kit SQK-108 and a PCR-free “native barcoding” kit according to the manufacturer’s protocol. Barcoded samples were pooled and loaded onto a MinION MIN106 flow cell controlled by MinKNOW version V1.2.8 software (ONT). Base calling was performed using albacore Basecall_Barcoding workflow (version 1.11.5) (ONT). The Fasta files of reads obtained from sequencing were subjected to a *de novo* assembler, Canu (https://github.com/marbl/canu), using default parameters. Assembled contigs were analyzed using sequence aligner Mauve (http://darlinglab.org/mauve/mauve.html) to find chromosomal rearrangements.

### *oriC* inversion prediction in E. coli genomes.

Six hundred seventy-five complete E. coli genomes downloaded from the NCBI ftp site were used for this analysis. For finding E. coli strains which possess a chromosomal inversion of the *oriC* region, blastn searching was performed on genomes with the E. coli K-12 MG1655 (NC_000913.3) genome as query and reference for inversion. The inverted regions from blast search output of complete genomes were stitched and added together to calculate the total inverted region; thus, an inference was made on the status of inversion of the region involving *oriC*. *oriC* positions in these genomes were predicted for all E. coli strains by performing blastn searching using the E. coli K-12 MG1655 (NC_000913.3) *oriC* region as query.

### *oriK* peak prediction.

*oriK* positions were predicted from the LOESS fitted copy number plots using custom R scripts. Outliers were removed by visualization from the copy number data before fitting the curve. The LOESS fit was derived after removing known deletions and reversing the copy number curve around inversions. A position was called as an *oriK* peak if it had a negative slope, measured relative to the peak position, up to 100 kbp in both directions in the LOESS predicted values. Peak range (mentioned in Table S5 in the supplemental material) is defined from the minimum to maximum position predicted for each peak site across strains.

### R-loop predictions using QmRLFs finder.

To predict RNA-DNA hybrids on the chromosome, we used the QmRLFs model (34, 35) on the Escherichia coli K-12 MG1655 (NC_000913.3) genome with default parameters. From the output file, we considered the starting position of a predicted R-loop and plotted a line plot for these positions using custom R scripts for both the models (m1 and m2) separately.

### RNA extraction, mRNA enrichment, and sequencing.

Overnight cultures were inoculated in 100 ml of fresh LB medium to bring the initial optical density (OD) of the culture to 0.03, and the flasks were incubated at 37°C with shaking at 200 rpm. Samples were collected at the maximum growth rate, and two biological replicates were performed for each sample. The samples were immediately processed for total RNA isolation using the TRIzol method (15596018; Invitrogen). DNase-treated RNA was depleted of rRNA using the Ambion MICROBExpress kit (AM1905). Libraries were prepared for RNA sequencing using the New England Biolabs (NEB) NextUltra directional RNA library prep kit for Illumina (New England Biolabs), according to the manufacturer’s protocol, and single-end sequencing for 50 cycles was done using the Illumina HiSeq 2500 platform.

### Transcriptome analysis.

The sequencing reads were aligned and mapped to the reference genome (NC_000913.3) using the Burrows-Wheeler aligner (BWA) (51). The reference genome sequence (.fna) and annotation (.gff) files for the same strain were downloaded from the NCBI ftp website (ftp://ftp.ncbi.nlm.nih.gov). The raw read quality was checked using the FastQC software (version v0.11.5). SAMtools (version 1.2) and BEDTools (version 2.25.0) were used to calculate the read count per gene using the annotation file (.bed). The format of the annotation file (.gff) was changed to .bed using an in-house python script. The normalization and differential gene expression analysis for the two conditions were carried out using the edgeR pipeline (54). Log fold change expression values in comparison to the *ΔrnhA-ΔdnaA* strain were plotted using in-house R scripts, and the Pearson correlation values were predicted for the same. The genes that are differentially expressed by a log(base 2) fold change of 1.5 or above with a false-discovery rate (FDR) value of 0.01 were considered differentially expressed.

### DNA copy number and transcriptome comparison.

The sequencing reads of both DNA and RNA isolated at the exponential phase of growth were analyzed similarly to the transcriptome analysis described above. The normalization and differential gene expression analysis for the two conditions were carried out using the edgeR (54) pipeline. Smoothed log fold change (logFC) expression values in comparison to the *ΔrnhA-ΔdnaA* strain were plotted against chromosome coordinates using in-house R scripts.

### Probability of head-on collision prediction.

The probability of head-on collisions in evolved and parental strains from RNA sequencing data was calculated for the chromosome region 3.3 Mb to 4.6 Mb, which includes the inversion. The rate of head-on collisions in the presence or absence of the inversion was calculated by assuming the activation of a single predominant origin of replication in evolved and parental clones (either *oriC* or *oriK45*). The fractional score of head-on replication-transcription conflicts was defined as the ratio of the number of reads mapping to genes carried on the lagging strand to the total number of reads mapping to the region for each strain. The strand information for genes was adapted from NC_000913 (version 3) .ptt or .rnt files.

### Promoter activity assay.

The promoter activity of the mutant and wild-type *rrnD* promoter region (*rrsD*-*yrdA* intergenic region [IGR]) was monitored by transforming the pUA139 plasmid containing the cloned construct of the IGR into wild-type E. coli. M9 medium with 0.2% glucose was used to culture the strains. Overnight culture containing the plasmid strain was inoculated at a ratio of 1:100 in 100 ml medium, and the samples were isolated at various intervals to measure GFP fluorescence using a FACSCalibur cytometer. Around 25,000 cells were acquired for each sample using a 488-nm excitation laser, and the emission was recorded from the FL1 channel, which uses a 530/30 band-pass (BP) filter to collect the GFP intensity The pUA139::*gfp* plasmid was used to set the background fluorescence, and GFP intensity above this background was marked as positive. Data were analyzed using FlowJo software.

### Data availability.

The genome sequence data from this work are available at NCBI BioProject (https://www.ncbi.nlm.nih.gov/bioproject/) under accession no. PRJNA562391. The RNA sequence data and processed files from this work are available at NCBI Geo (https://www.ncbi.nlm.nih.gov/geo/query/acc.cgi?acc) under accession no. GSE135706.

10.1128/mSphere.00939-19.10 TABLE S6 Strains, plasmids, and primers used in this study. Download Table S6, PDF file, 0.04 MB. Copyright © 2020 Veetil et al. 2020 Veetil et al. This content is distributed under the terms of the Creative Commons Attribution 4.0 International license.
