# Supplementary material for: Laboratory Evolution Experiments Help Identify a Predominant Region of Constitutive Stable DNA Replication Initiation
Source: mSphere. 2020 Feb 26;5(1):e00939-19. doi: 10.1128/mSphere.00939-19 (PMC7045392; doi:10.1128/mSphere.00939-19)
Supplement: TABLE S1 [file mSphere.00939-19-st001.pdf]

GCF_000008885	Wigglesworthia_glossinidia_endosymbiont_of_Glossina_brevipalpis_plasmid_pWb1_DNA
GCF_000011745	Candidatus_Blochmannia_pennsylvanicus_str._BPEN
GCF_000013185	Baumannia_cicadellinicola_str._Hc_(Homalodisca_coagulata)
GCF_000022605	Blattabacterium_sp._(Blattella_germanica)_str._Bge_plasmid_pBge
GCF_000025125	Candidatus_Atelocyanobacterium_thalassa_isolate_ALOHA
GCF_000043285	Blochmannia_floridanus_complete_genome
GCF_000093065	Candidatus_Riesia_pediculicola_USDA_plasmid_pPAN
GCF_000146025	Uncultured_Termite_group_1_bacterium_phylotype_Rs-D17_plasmid pTGRD3 DNA
GCF_000165505	Ilyobacter_polytropus_DSM_2926_plasmid_pILYOP02
GCF_000177535	Corynebacterium_resistens_DSM_45100
GCF_000179035	Mycoplasma_suis_str._Illinois
GCF_000185985	Candidatus_Blochmannia_vafer_str._BVAF
GCF_000196515	'Nostoc_azollae'_0708_plasmid_pAzo02
GCF_000203215	Mycoplasma_suis_KI3806_complete_genome
GCF_000219175	Candidatus_Moranella_endobia_PCIT
GCF_000223375	Ketogulonigenium_vulgarum_WSH-001_plasmid_2
GCF_000236405	Blattabacterium_sp._(Cryptocercus_punctulatus)_str._Cpu_plasmid_pCpu
GCF_000247565	Wigglesworthia_glossinidia_endosymbiont_of_Glossina_morsitans_morsitans
GCF_000255275	Corynebacterium_diphtheriae_PW8
GCF_000262655	Helicobacter_pylori_XZ274_plasmid_pXZ274
GCF_000287295	Candidatus_Carsonella_ruddii_HT_isolate_Thao2000
GCF_000292685	Candidatus_Portiera_aleyrodidarum_BT-B
GCF_000298385	Candidatus_Portiera_aleyrodidarum_BT-QVLC
GCF_000300035	Candidatus_Portiera_aleyrodidarum_BT-QVLC
GCF_000300075	Candidatus_Portiera_aleyrodidarum_BT-B

GCF_000304735	Borrelia_afzelii_HLJ01
GCF_000317675	Cyanobacterium_aponinum_PCC_10605_plasmid_pCYAN10605.01
GCF_000319385	Candidatus_Endolissoclinum_faulkneri_L2
GCF_000331065	Candidatus_Blochmannia_chromaiodes_str._640
GCF_000364725	Candidatus_Moranella_endobia_PCVAL
GCF_000441555	Candidatus_Profftella_armatura_plasmid
GCF_000471965	Blattabacterium_sp._(Nauphoeta_cinerea)_plasmid
GCF_000477415	Mycoplasma_parvum_str._Indiana
GCF_000505725	Francisella_noatunensis_subsp._orientalis_LADL--07-285A
GCF_000508245	Mycoplasma_ovis_str._Michigan
GCF_000604125	Treponema_pallidum_subsp._pallidum_str._Sea_81-4
GCF_000709555	Endosymbiont_of_Llaveia_axin_axin
GCF_000767685	Corynebacterium_ulcerans_FRC11
GCF_000769635	Corynebacterium_ulcerans_strain_05146
GCF_000770175	Mycobacterium_abscessus_strain_DJO-44274
GCF_000815025	Coxiella_endosymbiont_of_Amblyomma_americanum
GCF_000827855	Candidatus_Portiera_aleyrodidarum_MED_(Bemisia_tabaci)_strain_BT-Q
GCF_000828815	Candidatus_Tachikawaea_gelatinosa_DNA
GCF_000828835	Thioploca_ingrica_DNA
GCF_000829235	Cyanobacterium_endosymbiont_of_Epithemia_turgida_isolate_EtSB_Lake_Yunoko_DNA
GCF_000953435	Candidatus_Evansia_muelleri_genome_assembly_CEM1.1
GCF_000973545	Blochmannia_endosymbiont_of_Camponotus_(Colobopsis)_obliquus_strain_757
GCF_001021025	Corynebacterium_epidermidicanis_strain_DSM_45586
GCF_001278785	Candidatus_Profftella_armatura_strain_YCPA_plasmid
GCF_001318295	Candidatus_Xiphinematobacter_sp._Idaho_Grape
GCF_001548095	Geminocystis_sp._NIES-3708_plasmid_pGM05_DNA
GCF_001548115	Geminocystis_sp._NIES-3709_plasmid_pGM3709_11_DNA
GCF_001587015	Campylobacter_jejuni_strain_OD267_plasmid_pCJDM67_S

GCF_001682195	Flammeovirga_sp._MY04_plasmid
GCF_900048035	Enterobacteriaceae_bacterium_symbiont_of_Ferrisia_virgata_isolate_GEFVIR_genome_assembly
GCF_900048045	Enterobacteriaceae_bacterium_symbiont_of_Paracoccus_marginatus_isolate_MEPMAR_genome_assembly
