# Supplementary material for: Laboratory Evolution Experiments Help Identify a Predominant Region of Constitutive Stable DNA Replication Initiation
Source: mSphere. 2020 Feb 26;5(1):e00939-19. doi: 10.1128/mSphere.00939-19 (PMC7045392; doi:10.1128/mSphere.00939-19)
Supplement: TABLE S2 [file mSphere.00939-19-st002.pdf]

**Strain ID**	***Ori*-to-*ter* ratio**
Parental strains	
1D0_1	1.13
1D0_2	1
1D0_3	1.09
1D0_4	1.09
5D0_1	1.13
5D0_2	1.15
5D0_3	1.07
5D0_4	1.15
8D0_1	1.03
8D0_2	0.97
8D0_3	0.87
8D0_4	1
ΔrnhA-ΔdnaA	1.02
ΔrnhA	1.79
ΔrnhA-ΔdnaA/pHYD2388	1.22
GJ13519	2.48
K12 MG1655	2.35
Suppressor mutants	
1D4_1	1.02
1D4_2	1.73
1D4_3	1.69
1D4_4	1.03
1D8_1	1.51
1D8_2	1.45
1D8_3	0.94

1D8_4	1.93
1D12_2	1.28
1D12_3	1.27
1D12_4	1.35
1D15_1	1.42
1D15_2	1.49
1D15_3	1.25
1D15_4	1.47
5D4_1	1.41
5D4_2	1.33
5D4_3	1.38
5D4_4	1.20
5D8_1	1.34
5D8_2	1.38
5D8_3	1.47
5D8_4	1.31
5D12_1	1.68
5D12_2	1.82
5D12_3	2.68
5D12_4	1.67
5D15_1	1.82
5D15_2	1.88
5D15_3	1.89
5D15_4	1.96
8D4_1	1.14
8D4_2	1.02
8D4_3	1.03

8D4_4	1.07
8D8_1	1.44
8D8_2	1.87
8D8_3	1.76
8D8_4	1.36
8D15_1	1.70
8D15_2	1.70
8D15_4	1.72
