# Supplementary material for: Laboratory Evolution Experiments Help Identify a Predominant Region of Constitutive Stable DNA Replication Initiation
Source: mSphere. 2020 Feb 26;5(1):e00939-19. doi: 10.1128/mSphere.00939-19 (PMC7045392; doi:10.1128/mSphere.00939-19)
Supplement: TABLE S4 [file mSphere.00939-19-st004.pdf]

Sample	3.84-3.89 (*oriC*)	0.44-0.55	1.42-1.52	1.88-2.23	2.53-2.6	2.95-3.36	3.63-3.8
**Parental strains**							
WT K12	2.36	-	-	-	-	-	-
GJ13519	2.5	-	-	-	-	-	-
ΔrnhA	1.9	-	1.18	-	-	-	-
ΔdnaA/dnaA	2.33	-	-	-	-	-	-
ΔrnhA-ΔdnaA/dnaA	-	1.33	1.22	1.17	1.1	-	1.21
ΔrnhA-ΔdnaA	-	1.63	1.85	1.5	-	1.19	1.-5
1D0_1	-	1.35	1.33	1.27	1.15	1.17	-
1D0_2	-	1.53	1.74	1.5	-	1.21	1.-7
1D0_3	-	1.56	1.64	1.44	1.22	1.18	1.-7
1D0_4	-	1.34	1.34	1.41	1.32	1.36	1.-8
5D0_1	-	1.47	1.49	1.37	1.2	1.18	1.-4
5D0_2	-	1.56	1.56	1.4	1.24	1.2	-
5D0_3	-	1.63	1.73	-	-	1.25	1.-9
5D0_4	-	1.56	1.55	1.43	1.25	1.21	1.-7
8D0_1	-	1.33	1.47	1.26	1.11	1.-8	1.-2
8D0_2	-	1.5	1.76	1.42	1.26	1.18	1.-4
8D0_3	-	1.31	1.68	1.31	1.-5	1.-6	1.-2
8D0_4	-	1.48	1.7	1.42	-	1.19	1.-5
**Suppressor strains**							
1D4_1	-	1.22	1.41	1.35	1.31	1.35	1.25
1D4_4	-	-	1.6	1.51	-	-	1.-4
1D8_3	-	-	1.83	1.-9	-	1.24	1.11
5D4_1	-	-	1.3	1.18	-	-	-
5D4_2	-	-	1.28	1.15	-	-	1.34
5D4_3	-	-	1.29	1.16	-	-	1.38
5D4_4	-	-	1.29	1.12	-	-	1.24
5D8_1	-	-	1.29	1.16	-	-	-
5D8_2	-	-	1.28	1.15	-	-	1.38
5D8_3	-	-	1.32	1.19	-	-	-

5D8_4	-	-	1.27	1.15	-	-	1.32
5D12_1	-	-	1.51	1.-9	-	-	-
5D12_2	-	1.8	1.39	1.-9	1.11	1.25	1.29
5D12_3	-	-	1.28	-	-	-	-
5D12_4	-	-	1.61	1.-7	-	1.3	1.36
5D15_1	-	-	1.41	1.17	-	-	-
5D15_2	-	-	1.44	1.19	-	-	1.75
5D15_3	-	-	1.42	1.2	-	-	1.74
5D15_4	-	-	1.43	1.25	-	-	-
8D4_1	-	-	1.1	1.14	-	1.18	-
8D4_2	-	1.2	1.38	1.36	1.36	-	1.-6
8D4_3	-	-	1.49	1.46	-	1.44	-
8D4_4	-	-	1.34	1.35	1.41	-	-
1D4_2	-	-	1.41	1.26	-	1.58	-
1D4_3	-	-	1.4	1.25	-	-	-
1D8_1	-	-	1.63	1.18	-	-	-
1D8_4	-	-	-	1.3	-	-	-
1D8_2	-	-	1.32	1.18	-	-	-
1D12_2	-	-	1.23	1.16	-	1.34	1.28
1D12_3	-	-	1.35	1.13	-	-	-
1D12_4	-	-	1.39	1.18	-	-	-
1D15_1	-	-	1.24	1.19	-	-	1.38
1D15_2	-	-	1.31	1.21	-	-	-
1D15_3	-	-	1.49	1.13	-	-	-
1D15_4	-	1.57	1.29	1.22	-	-	-
8D8_1	-	-	1.54	1.1	-	-	-
8D8_2	-	-	1.35	1.21	-	-	-
8D8_3	-	-	1.37	1.18	-	-	-
8D8_4	-	-	1.55	1.-6	-	-	-
8D15_1	-	-	1.37	1.14	-	-	-
8D15_2	-	-	1.34	1.15	-	-	-
8D15_4	-	-	1.33	-	-	-	-
