# Supplementary material for: Laboratory Evolution Experiments Help Identify a Predominant Region of Constitutive Stable DNA Replication Initiation
Source: mSphere. 2020 Feb 26;5(1):e00939-19. doi: 10.1128/mSphere.00939-19 (PMC7045392; doi:10.1128/mSphere.00939-19)
Supplement: TABLE S5 [file mSphere.00939-19-st005.pdf]

ΔrnhA Downregulated genes					
COG categories	COG & DE	COG but !DE	!COG but DE	!COG&!DE	P-value
**CELLULAR PROCESSES AND SIGNALING**					
[D] Cell cycle control, cell division	1	32	338	3557	0.35
[M] Cell wall/ membrane/ envelope biogenesis	12	216	327	3373	0.06
[N] Cell motility	27	84	312	3505	3.94x10-7*
[O] Post-translational modification	17	124	322	3465	0.1664
[T] Signal transduction mechanisms	14	164	325	3425	0.89
[U] Intracellular trafficking and secretion	12	115	327	3474	0.74
[V] Defense mechanisms	3	46	336	3543	0.79
**INFORMATION STORAGE AND PROCESSING**					
[J] Translation	8	176	331	3413	0.03
[K] Transcription	17	286	322	3303	0.05
[L] Replication, recombination and repair	6	199	333	3390	0.001
**METABOLISM**					
[C] Energy production and conversion	42	245	297	3344	0.0004*
[E] Amino acid transport and metabolism	34	328	305	3261	0.55
[F] Nucleotide transport and metabolism	8	90	331	3499	1
[G] Carbohydrate transport and metabolism	61	308	278	3281	2.48x10-7*
[H] Coenzyme transport and metabolism	4	152	335	3437	0.003
[I] Lipid transport and metabolism	5	95	334	3494	0.27
[P] Inorganic ion transport and metabolism	12	203	327	3386	0.1
[Q] Secondary metabolites biosynthesis	4	60	335	3529	0.65
**POORLY CHARACTERIZED**					

[R] General function prediction only	29	372	310	3217	0.34
[S] Function unknown	23	294	316	3295	0.4

**ΔrnhA Upregulated genes**					
COG categories	COG & DE	COG but !DE	!COG but DE	!COG&! DE	P-value
**CELLULAR PROCESSES AND SIGNALING**					
[D] Cell cycle control, cell division	3	30	345	3550	1
[M] Cell wall/ membrane/ envelope biogenesis	10	218	338	3362	0.011
[N] Cell motility	13	98	335	3482	0.3
[O] Post-translational modification	9	132	339	3448	0.36
[T] Signal transduction mechanisms	12	166	336	3414	0.34
[U] Intracellular trafficking and secretion	15	112	333	3468	0.26
[V] Defense mechanisms	1	48	347	3532	0.12
**INFORMATION STORAGE AND PROCESSING**					
[J] Translation	35	149	313	3431	1.244X10 -5
[K] Transcription	32	271	316	3309	0.29
[L] Replication, recombination and repair	20	185	328	3395	0.61
**METABOLISM**					
[C] Energy production and conversion	41	246	307	3334	0.0016
[E] Amino acid transport and metabolism	32	330	316	3250	1
[F] Nucleotide transport and metabolism	9	89	339	3491	0.85
[G] Carbohydrate transport and metabolism	29	340	319	3240	0.56
[H] Coenzyme transport and metabolism	7	149	341	3431	0.059
[I] Lipid transport and metabolism	6	94	342	3486	0.37
[P] Inorganic ion transport and metabolism	27	188	321	3392	0.06

[Q] Secondary metabolites biosynthesis	6	58	342	3522	0.82
**POORLY CHARACTERIZED**					
[R] General function prediction only	28	373	320	3207	0.19
[S] Function unknown	13	304	335	3276	0,00092

**ΔrnhA-ΔdnaA Down-regulated genes**					
COG categories	COG & DE	COG but !DE	!COG but DE	!COG&! DE	P-value
**CELLULAR PROCESSES AND SIGNALING**					
[D] Cell cycle control, cell division	2	31	514	3381	0.3
[M] Cell wall/ membrane/ envelope biogenesis	17	211	499	3201	0.008
[N] Cell motility	29	82	487	3330	0.00016
[O] Post-translational modification	24	117	492	3295	0.16
[T] Signal transduction mechanisms	22	156	494	3256	0.8
[U] Intracellular trafficking and secretion	15	112	501	3300	0.78
[V] Defense mechanisms	8	41	508	3371	0.52
**INFORMATION STORAGE AND PROCESSING**					
[J] Translation	10	174	506	3238	0.0007
[K] Transcription	34	269	482	3143	0.33
[L] Replication, recombination and repair	14	191	502	3221	0.004
**METABOLISM**					
[C] Energy production and conversion	62	225	454	3187	3.71x10-5
[E] Amino acid transport and metabolism	46	316	470	3096	0.87
[F] Nucleotide transport and metabolism	11	87	505	3325	0.65
[G] Carbohydrate transport and metabolism	82	287	434	3125	3.92x10-7
[H] Coenzyme transport and metabolism	10	146	506	3266	0.01
[I] Lipid transport and	8	92	508	3320	0.135

metabolism					
[P] Inorganic ion transport and metabolism	22	193	494	3219	0.2
[Q] Secondary metabolites biosynthesis	6	58	510	3354	0.45
**POORLY CHARACTERIZED**					
[R] General function prediction only	60	341	456	3071	0.27
[S] Function unknown	34	283	482	3129	0.19

**ΔrnhA-ΔdnaA Up-regulated genes**					
COG categories	COG & DE	COG but !DE	!COG but DE	!COG&! DE	P-value
**CELLULAR PROCESSES AND SIGNALING**					
[D] Cell cycle control, cell division	2	33	425	3468	0.58
[M] Cell wall/ membrane/ envelope biogenesis	23	205	404	3296	0.82
[N] Cell motility	10	101	417	3400	0.64
[O] Post-translational modification	12	129	415	3372	0.41
[T] Signal transduction mechanisms	18	160	409	3341	0.9
[U] Intracellular trafficking and secretion	11	116	416	3385	0.47
[V] Defense mechanisms	0	47	427	3454	0.007
**INFORMATION STORAGE AND PROCESSING**					
[J] Translation	45	139	382	3362	7.11E-08
[K] Transcription	31	272	396	3229	0.77
[L] Replication, recombination and repair	31	174	396	3327	0.04986
**METABOLISM**					
[C] Energy production and conversion	27	260	400	3241	0.49
[E] Amino acid transport and metabolism	50	312	377	3189	0.06
[F] Nucleotide transport and metabolism	14	84	413	3417	0.25
[G] Carbohydrate transport and metabolism	19	350	408	3151	9.697X10 -5

[H] Coenzyme transport and metabolism	13	143	414	3358	0.7
[I] Lipid transport and metabolism	8	92	419	3409	0.41
[P] Inorganic ion transport and metabolism	39	176	388	3325	0.000981
[Q] Secondary metabolites biosynthesis	11	53	416	3448	0.1
**POORLY CHARACTERIZED**					
[R] General function prediction only	38	363	389	3138	0.39
[S] Function unknown	25	292	402	3209	0.08
