# Supplementary material for: Laboratory Evolution Experiments Help Identify a Predominant Region of Constitutive Stable DNA Replication Initiation
Source: mSphere. 2020 Feb 26;5(1):e00939-19. doi: 10.1128/mSphere.00939-19 (PMC7045392; doi:10.1128/mSphere.00939-19)
Supplement: TABLE S6 [file mSphere.00939-19-st006.pdf]

**Strain**	**Genotype**	**Source**
GJ13519	MG1655 Δ(argF-lac)U169	LBG, CDFD
**Derivatives of GJ13519**		
ΔrnhA	Δ*rnhA*::FRT	LBG, CDFD
ΔdnaA/dnaA$^+$	*ΔdnaA*::FRT/pHYD2388	LBG, CDFD
ΔrnhA-dnaA/dnaA$^+$	*ΔdnaA*::FRT *ΔrnhA*::FRT/pHYD2388	LBG, CDFD
ΔrnhA-ΔdnaA	ΔdnaA::FRT ΔrnhA::FRT	This study
GJ13519/dnaA$^+$	GJ13519/pHYD2388	This study
GJ13519ΔhotH/dnaA$^+$	GJ13519 Δ/pHYD2388	This study
ΔrnhAΔdnaAΔhotH/dnaA$^+$	*ΔdnaA*::FRT *ΔrnhA*::FRT Δ4555284: 45660615(uxuR-yjiN) /pHYD2388	This study
ΔrnhA-ΔdnaA-ΔrnhA-ΔdnaAΔhotH	*ΔdnaA*::FRT *ΔrnhA*::FRT ΔΔ4555284: 45660615(uxuR-yjiN)	This study
**Plasmid**	**Description**	**Source**
pUA139	Low copy plasmid with fast folding GFP mut2	SAFS lab
pUA139::Wt rrnD IGR	pUA139 vector carrying 598bp *rrsD-yrdA* intergenic region sequence	This study
pUA139::Mut rrnD IGR	pUA139 vector carrying 598bp rrsD-yrdA mutant intergenic [G-A(3,429,052), +A(3,429,054)] sequence	This study
pHYD2388	*pMU575* derivative carrying *S.enterica dnaA*+	LBG, CDFD
**Primer Description**	**Sequence 5' -----> 3'**	**Source**
*HotH*KO *pKD13* F	GTTGACGATATTTATTTTGATGGCTATCTGTTTGA Tgtgtaggctggagctgcttcg	This study
*HotH*KO *pKD13* R	TTCGCTGGCTGGAGAGCGAGCATCCACTGAAAG CCAattccggggatccgtcgacc	This study
rrsD -yrdA IGR F	ATTACTCGAGTCGTCAGCGAAACAGCAA	This study
rrsD-yrdA IGR R	TAATAGATCTGTATGGGCGTAAAACATC	This study
